# The Effect of Lumbar Multifidus and Transversus Abdominis Muscle Activation on Balance in Patients with Hemiplegia

**DOI:** 10.3390/jcm15155763

**Published:** 2026-07-23

**Authors:** Emre Erol, Derya Bugdayci, Mert Cetin, Eser Kalaoglu

**Affiliations:** 1 Department of Physical Medicine and Rehabilitation, Istanbul Physical Therapy and Rehabilitation Education and Research Hospital, 34186 Istanbul, Türkiye; deryabugdayci@yahoo.com (D.B.); eserkalaoglu@hotmail.com (E.K.); 2 Department of Physical Medicine and Rehabilitation, Faculty of Medicine, Yeditepe University, 34755 Istanbul, Türkiye; drmertcetin@gmail.com

**Keywords:** stroke, hemiplegia, balance, transversus abdominis, multifidus

## Abstract

**Background/Objectives:** This study investigated whether ultrasonographically assessed contractility of the transversus abdominis (TrA) and lumbar multifidus muscles predicts balance and postural control in stroke-related hemiplegia. **Methods:** This cross-sectional study included 36 patients with subacute or chronic stroke. Balance was assessed using the Berg Balance Scale (BBS) and the Postural Assessment Scale for Stroke Patients (PASS). Bilateral L2–3, L3–4, and L4–5 multifidus and bilateral TrA muscle thickness were measured ultrasonographically at rest and during contraction. The thickening fraction (TF, %), resting thickness ratio (RTR, %), and TF ratio (TFR, %) were calculated. Significant correlates were entered into hierarchical regression models to identify independent predictors of balance. **Results:** The mean BBS was 39.92 ± 13.67, and the mean PASS-Total was 30.06 ± 4.59 (Maintaining a Posture [MP]: 10.65 ± 2.56; Changing a Posture [CP]: 19.41 ± 2.52). Moderate positive correlations were found between paretic-side L2–3 (pL2–3) multifidus TF and BBS and PASS-MP, while weak positive correlations were observed between paretic-side TrA TF and PASS-CP and between TrA TFR and BBS, PASS-Total, and PASS-CP. In hierarchical regression models, pL2–3 multifidus TF was a significant independent predictor of both BBS (ΔR^2^ = 0.102, *p* = 0.020, β = 0.340) and PASS-MP (ΔR^2^ = 0.090, *p* = 0.020, β = 0.320). Muscle parameters did not independently predict PASS-Total or PASS-CP. **Conclusions:** Paretic-side lumbar multifidus activation was identified as a significant independent predictor of balance and postural control, whereas TrA parameters did not independently predict balance outcomes in this population.

## 1. Introduction

Stroke is the second leading cause of death and the third leading cause of disability-adjusted life years (DALYs) among non-communicable diseases worldwide [1]. Following a cerebrovascular event, significant impairments in trunk control and balance may occur due to motor control deficits, spasticity, and proprioceptive dysfunction [2,3]. In this context, deep trunk muscles—specifically, the transversus abdominis (TrA) and lumbar multifidus—play a critical role [4,5,6].

Trunk muscles are essential for maintaining posture, enabling limb movement against gravity, and performing activities of daily living (ADLs) [7]. Unlike the extremities, trunk muscles are innervated bilaterally; therefore, a unilateral stroke can impair trunk muscle function on both the contralateral and ipsilateral sides [8]. This impairment may lead to loss of balance, increased postural sway, and a higher risk of falls in patients with hemiplegia [9,10].

Previous studies have reported significant associations between static balance and hospital length of stay, musculoskeletal function, and post-stroke functional abilities [11,12,13,14,15]. Additionally, trunk muscle control, particularly in the early post-stroke period, has been shown to be a strong predictor of functional improvement in ADLs over the subsequent six months [16]. Rehabilitation programs incorporating trunk stabilization exercises (TSEs) have also demonstrated positive effects on balance, gait, and ADLs [17,18].

However, the current literature has primarily examined the relationship between trunk muscles and balance at the correlational level, and studies demonstrating their independent predictive contribution to functional balance remain limited [19,20,21]. Furthermore, the lumbar multifidus muscle has often been evaluated globally, whereas segmental-level investigations are scarce. Therefore, the aim of this study was to investigate the predictive role of TrA and lumbar multifidus muscle thickness and thickening fraction on functional balance and postural control in patients with hemiplegia.

## 2. Materials and Methods

### 2.1. Participants

This study was conducted between November 2022 and October 2023 at the physical medicine and rehabilitation unit of a tertiary training and research hospital in Türkiye. Patients who received inpatient treatment during this period were recruited.

The study was approved by the local ethics committee (No: 2022-22-15, 21 November 2022) and conducted in accordance with the Declaration of Helsinki. Written informed consent was obtained from all participants. The study was registered at ClinicalTrials.gov (NCT06181877). This observational study was reported in accordance with the Strengthening the Reporting of Observational Studies in Epidemiology (STROBE) guidelines.

#### 2.1.1. Inclusion Criteria

- Age 18–65 years;
- At least 1 month post stroke;
- History of unilateral stroke;
- Ability to cooperate cognitively;
- Ability to maintain balance while sitting without support and/or balance while standing with or without support;
- BMI < 30 kg/m^2^.

#### 2.1.2. Exclusion Criteria

- History of hemiplegia on the opposite side of the body;
- Diagnosis of extruded or sequestrated lumbar herniation and/or a history of lumbar surgery;
- History of lumbar vertebral fracture;
- Traumatic brain injury;
- Neuromuscular disorders that may cause muscle atrophy;
- Sensory deficits;
- Cerebellar sequelae;
- Vestibular balance disorders (e.g., vertigo);
- Parkinson’s disease;
- Balance impairments due to visual deficits;
- Pre-stroke balance disorders.

### 2.2. Study Method

In this cross-sectional study, demographic and anthropometric characteristics, clinical stroke features, and balance status were assessed. All ultrasonographic muscle thickness measurements were performed by the same researcher under standardized conditions on both the paretic and non-paretic sides.

#### 2.2.1. Outcome Variables

##### Primary Outcome Variables

- Berg Balance Scale (BBS): A 14-item scale used to assess static and dynamic balance and estimate fall risk. Each item is scored from 0 to 4, with a maximum total score of 56. A score of 0 indicates inability to perform the task, while a score of 4 indicates independent and safe performance. The Turkish validity and reliability of the scale has been established [22].
- Postural Assessment Scale for Stroke Patients—Total Score (PASS-Total): This scale comprises two subscales—Maintaining a Posture (five items) and Changing a Posture (seven items)—with a total of 12 items. Each item is scored on a scale of 0–3, with a maximum total score of 36. Higher PASS-Total scores indicate better postural control, reflecting an improved ability to maintain static positions and perform dynamic postural tasks in stroke patients. The Turkish validity and reliability of the scale have been established for patients with stroke [23].
- PASS—Maintaining a Posture (PASS-MP) Subscale: This subscale assesses the ability to maintain static postural positions (e.g., sitting and standing), with higher scores indicating better static postural control.
- PASS—Changing a Posture (PASS-CP) Subscale: This subscale assesses the ability to change and transition between postural positions, with higher scores indicating greater dynamic postural mobility.

##### Secondary Outcome Variables

- Ultrasonographic (USG) Thickness and Contractility Parameters of the Lumbar Multifidus and TrA Muscles: Muscle thickness was measured at rest and during contraction (mm). Measurements were repeated twice, and the mean value was recorded. The thickening fraction (TF, %) was calculated as the difference between the contracted and resting thickness divided by the resting thickness. The resting thickness ratio (RTR, %) and thickening fraction ratio (TFR, %) were calculated to eliminate individual differences and quantify bilateral asymmetry, using each patient’s non-paretic side as an internal reference. The resting thickness ratio was calculated by dividing the paretic-side resting thickness by the non-paretic side resting thickness. The TFR was calculated by dividing the paretic-side TF by the non-paretic-side TF. All USG parameters (RTR, TF, and TFR) were calculated as percentages (×100) and treated as continuous variables in all analyses.
- Brunnstrom Stage of Recovery (BRS): Used to assess motor recovery in patients with post-stroke hemiplegia. It comprises upper extremity (UE), hand, and lower extremity (LE) subscales, each of which is scored from 1 to 6. The BRS–Hand was excluded from the analyses for two reasons. First, the high correlation between BRS–UE and BRS–Hand poses a risk of multicollinearity in the regression analysis. Second, a review of the literature on balance in patients with hemiplegia indicates that upper extremity motor function scales, such as the BRS–UE or the Fugl–Meyer Assessment–Upper Extremity (FMA–UE), are widely used [24,25], whereas the hand is considered to influence balance primarily through sensory perception rather than its motor function [26].

#### 2.2.2. Ultrasonographic Muscle Measurements

**Figure 1:**
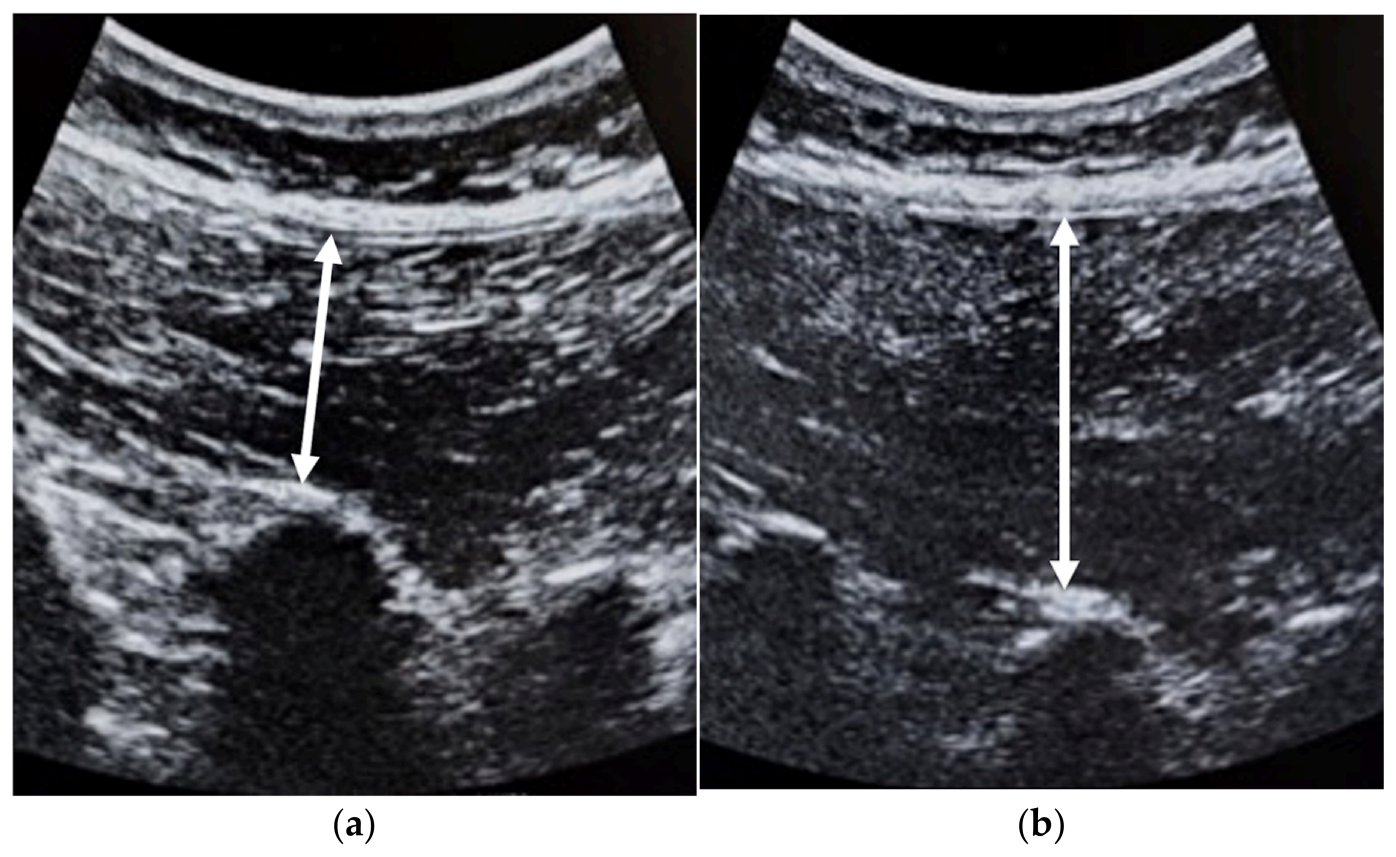
Lumbar multifidus thickness measurements: (**a**) resting; (**b**) contraction. Double-headed arrows indicate the measurement of muscle thickness at rest (**a**) and during contraction (**b**).

For multifidus measurements, the patients were positioned prone with a pillow under the abdomen to reduce lumbar lordosis. Muscle thickness was measured as the vertical distance between the apex of the facet joint and the inner border of the thoracolumbar fascia. Since severe lower extremity motor deficits and asymmetric trunk control in some patients precluded the standard submaximal contralateral leg/arm raise protocol, a modified approach was adopted: the patients performed maximal trunk extension to the extent tolerated, based on the established role of the multifidus as a primary lumbar extensor [27]. The probe was positioned 2–3 cm lateral to the spinous processes, parallel to the spinal axis. Beginning at the sacrum, the probe was moved cranially and resting thickness was recorded at the L4–5 facet joint level, followed by contracted thickness during maximal lumbar extension at the same site. To minimize artifacts from spinal movement and skin sliding, the facet joint reference was tracked dynamically and the probe angle was maintained perpendicular (90°) to the fascia and muscle fibers throughout. Measurements were then repeated at the L3–4 and L2–3 levels (Figure 1).

**Figure 2:**
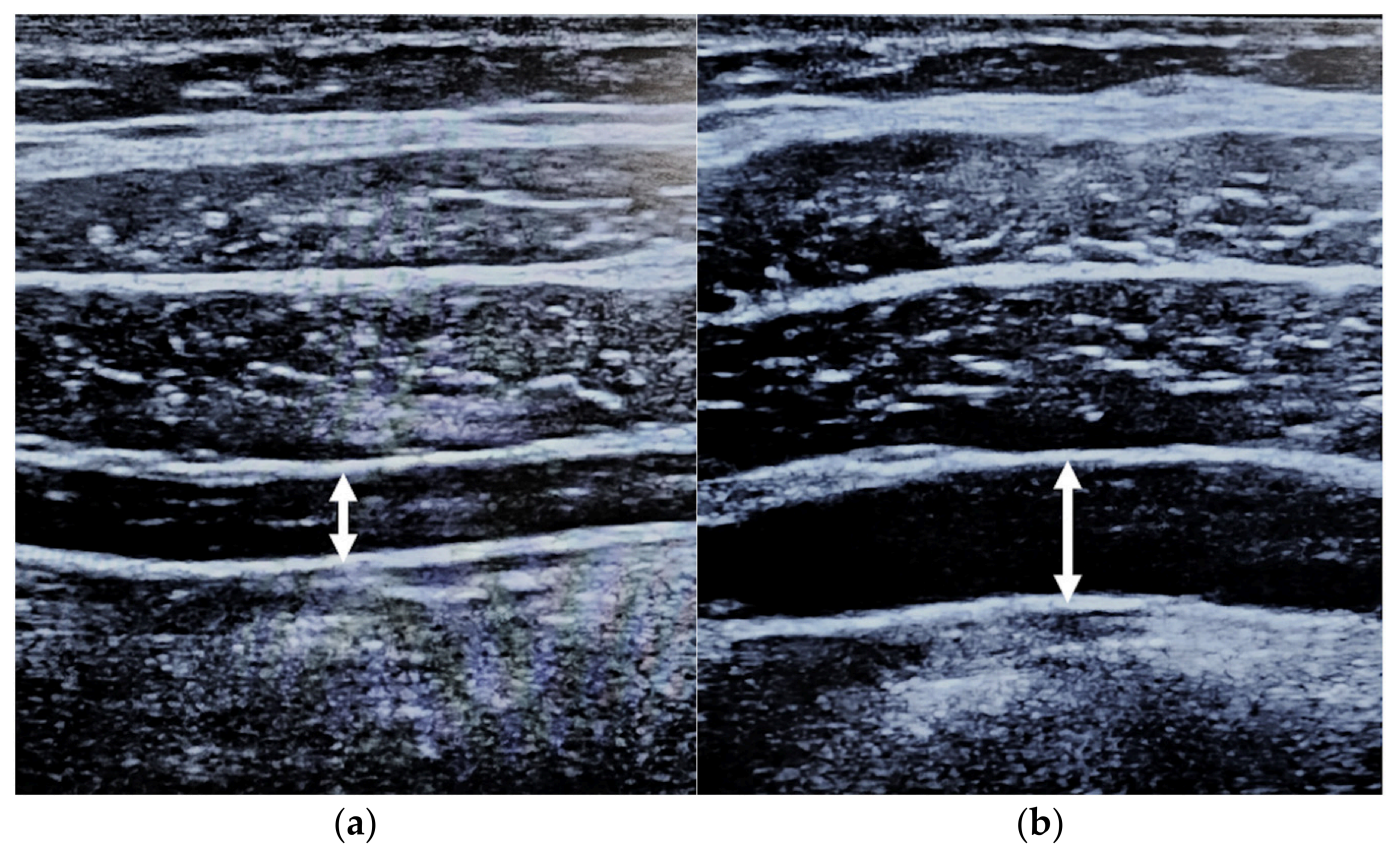
Transversus abdominis thickness measurements: (**a**) resting; (**b**) contraction. Double-headed arrows indicate the measurement of muscle thickness at rest (**a**) and during contraction (**b**).

The probe was placed approximately 2 cm medial to the mid-axillary line, between the last rib and the iliac crest, and the TrA thickness was measured as the perpendicular distance between the lower border of the internal oblique (IO) and the upper border of the transversalis fascia at rest and during the abdominal drawing-in maneuver (ADIM) (Figure 2).

All measurements were performed using an ultrasound device (DC-7, Mindray Bio-Medical Electronics, Shenzhen, China) with a 5–10 MHz linear probe for the TrA and a 3.8–5 MHz convex probe for the multifidus.

Ultrasonographic assessments were performed by a single assessor blinded to the balance evaluation scores, whereas functional balance and postural control evaluations were conducted by a separate assessor blinded to the ultrasonographic findings.

### 2.3. Sample Size Calculation

The sample sizes were calculated separately for the correlation and regression analyses. For the correlation analysis, the correlation coefficient (r = 0.53) identified by Kim et al. between trunk muscle contraction ratios and the BBS in stroke patients was used as a reference [4]. In the a priori power analysis conducted using G*Power software (version 3.1.9.7; Heinrich Heine University Düsseldorf, Düsseldorf, Germany), the sample size was calculated as 29 for a medium-to-large effect size (r = 0.53, α = 0.05, power = 0.80). Since no regression analysis linking ultrasonographic measurements of trunk muscles to balance outcomes has been reported in the literature, the correlation coefficient (r = 0.53) reported by Kim et al. was also used as a reference for the hierarchical regression models [4]. In the power analysis conducted using G*Power, a model with three predictors was determined to require at least 33 participants for a large effect size (f^2^ = 0.39, α = 0.05, power = 0.80). Based on the criterion of at least 10 observations per predictor, the sample size for a regression model with three predictors was calculated to be at least 30 [28]. To satisfy all criteria, 36 patients were enrolled in the study.

### 2.4. Statistical Analysis

Data were analyzed using IBM SPSS Statistics for Windows, version 22.0 (IBM Corp., Armonk, NY, USA). Normality was assessed using the Shapiro–Wilk test. Continuous variables were expressed as mean ± standard deviation (SD), ordinal variables as median and interquartile range (Q1–Q3), and categorical variables as frequencies and percentages. A paired-samples t-test was used to compare the muscle parameters between the paretic and non-paretic sides. The relationships between the dependent and independent variables were examined using Pearson (r) or Spearman correlation coefficients (r_s_), depending on the distribution of each variable, as assessed by the Shapiro–Wilk test. Correlation coefficients (r or r_s_) greater than 0.25 with *p* < 0.05 were considered clinically meaningful, consistent with a small-to-medium effect size threshold [29], and were included as candidates for the hierarchical regression analyses. Correlation analyses were performed as an exploratory step to identify potential predictors for the hierarchical regression analyses; therefore, no correction for multiple comparisons was applied. To assess the robustness of these exploratory findings, a post hoc sensitivity analysis incorporating a Bonferroni correction was subsequently performed on the significant exploratory correlations. This analysis sought to determine whether these associations retained their robustness after accounting for multiple comparisons (Type I error), as outlined in Section Sensitivity Analysis for Multiple Comparisons. Non-muscle parameters known to influence balance were entered in Block 1 as control variables, and muscle parameters were entered in Block 2 to evaluate their incremental contribution to the model.

Age, a well-known correlate of balance, was included as a control variable. To address potential multicollinearity arising from the high correlation between BRS–UE and BRS–LE, only the BRS stage demonstrating the stronger correlation with the dependent variable was retained in Block 1. The BRS, an ordinal variable, was treated as a continuous covariate and entered directly into the regression models without further transformation. All continuous variables were retained in their original forms without categorization. To ensure methodological transparency, preliminary hierarchical regression analyses examining the individual and combined effects of both BRS stages on the dependent variables are presented in the Supplementary Materials. Model assumptions were evaluated using the Variance Inflation Factor (VIF) for multicollinearity and the Durbin–Watson statistic for autocorrelation; normality of residuals was visually examined using Q-Q plots.

Correlation analyses among the dependent variables and between the dependent variables and BRS stages, as well as regression results for muscle parameters that did not reach statistical significance, are provided in the Supplementary Materials for methodological transparency.

No missing data were found for any of the variables in this study.

Measurement reliability was assessed using the intraclass correlation coefficient (ICC), based on repeated measurements performed by the same rater on 10 healthy individuals at a 48 h interval. The ICC(3,1) was calculated using a two-way mixed-effects model with absolute agreement. Reliability was interpreted as follows: ICC < 0.50, poor; 0.50–0.75, moderate; 0.75–0.90, good; and >0.90, excellent [30].

## 3. Results

Thirty-six patients with hemiplegia (23 men and 13 women) who met the inclusion and exclusion criteria were enrolled. The mean age was 57.19 ± 5.52 years, the mean time since stroke onset was 17.64 ± 23.14 months, and the mean BMI was 26.00 ± 2.96 kg/m^2^.

The mean BBS score was 39.92 ± 13.67, and the mean PASS-Total score was 30.06 ± 4.59 (PASS-MP: 10.65 ± 2.56; PASS-CP: 19.41 ± 2.52). The median BRS–UE stage was 4 (Q1–Q3: 3–5), and the median BRS–LE stage was 4 (Q1–Q3: 3–5).

A comparison of muscle values measured by USG between the paretic and non-paretic sides is presented in Table 1. Paretic-side TrA contracted thickness and TF were significantly lower than those on the non-paretic side, while the L4–5 multifidus TF was also significantly lower on the paretic-side. No statistically significant differences were observed between the two sides at other multifidus levels.

### 3.1. Correlation Analyses

Moderate positive correlations were found between BBS and both BRS–UE (r_s_ = 0.594, *p* < 0.001) and BRS–LE (r_s_ = 0.573, *p* < 0.001). A moderate positive correlation was also observed with paretic-side L2–3 (pL2–3) multifidus TF, and a weak positive correlation with TrA TFR (r_s_ = 0.376, *p* = 0.024). A moderate negative correlation was found with age (r_s_ = −0.467, *p* = 0.004).

Moderate positive correlations were found between PASS-Total and both BRS–UE (r_s_ = 0.595, *p* < 0.001) and BRS–LE (r_s_ = 0.580, *p* < 0.001). A weak negative correlation was observed with age (r_s_ = −0.380, *p* = 0.022) and a weak positive correlation with TrA TFR (r_s_ = 0.370, *p* = 0.027).

Moderate positive correlations were found between PASS-MP and both BRS–UE (r_s_ = 0.559, *p* < 0.001) and BRS–LE (r_s_ = 0.552, *p* < 0.001). A moderate negative correlation was observed with age (r_s_ = −0.513, *p* = 0.001). A moderate positive correlation was also found with pL2–3 multifidus TF (r_s_ = 0.583, *p* < 0.001).

Moderate positive correlations were found between PASS-CP and both BRS–UE (r_s_ = 0.489, *p* = 0.002) and BRS–LE stages (r_s_ = 0.428, *p* = 0.009). Additionally, weak positive correlations were observed with paretic-side TrA (pTrA) TF (r_s_ = 0.347, *p* = 0.038) and TrA TFR (r_s_ = 0.379, *p* = 0.022). The correlation analysis of all variables is presented in Table 2.

The correlations among the dependent variables and between the dependent variables and the BRS stages are presented in Supplementary Table S1.

#### Sensitivity Analysis for Multiple Comparisons

In light of the 60 correlations tested between 15 independent and 4 dependent variables, a post hoc Bonferroni correction was employed as a sensitivity analysis, setting α at 0.00083 (0.05/60). Among the 17 correlations initially significant at the uncorrected level, 8 retained significance following correction (all *p* < 0.001). These included correlations of BRS-UE with BBS, PASS-Total, and PASS-MP; BRS-LE with BBS, PASS-Total, and PASS-MP; and pL2–3 multifidus TF with BBS and PASS-MP. The remaining 9 correlations did not withstand the correction, such as BRS-UE and BRS-LE with PASS-CP (*n* = 2), age with balance outcomes (*n* = 3), and other muscle parameters that did not independently contribute to the regression models (*n* = 4). Consequently, these results should be interpreted with caution.

### 3.2. Regression Analyses

#### 3.2.1. BBS Regression Model

For the BBS model, BRS–UE (r = 0.594, *p* < 0.001) was included as a control variable in Block 1 of the hierarchical regression, as it demonstrated a higher correlation with BBS scores than the BRS–LE (r = 0.573, *p* < 0.001). The univariate and combined regression models for BRS–UE and BRS–LE are presented in Supplementary Table S2. Furthermore, age, a well-established factor associated with balance, was included as an additional control variable in Block 1, along with BRS–UE.

Block 1 of the hierarchical regression model, which included BRS–UE and age as control variables, explained 31.8% of the variance in BBS scores (R = 0.597, R^2^ = 0.357, adjusted R^2^ = 0.318, F(2,33) = 9.16, *p* < 0.001).

In the second block, pL2–3 multifidus TF and TrA TFR, which had demonstrated moderate and weak positive correlations with BBS, respectively, were entered as predictors. TrA TFR (β = 0.192, *p* = 0.192) did not make a statistically significant contribution to the model and was therefore excluded from the main model (ΔR^2^ = 0.031, F change = 1.63, *p* = 0.210) (Supplementary Table S3).

**Figure 3:**
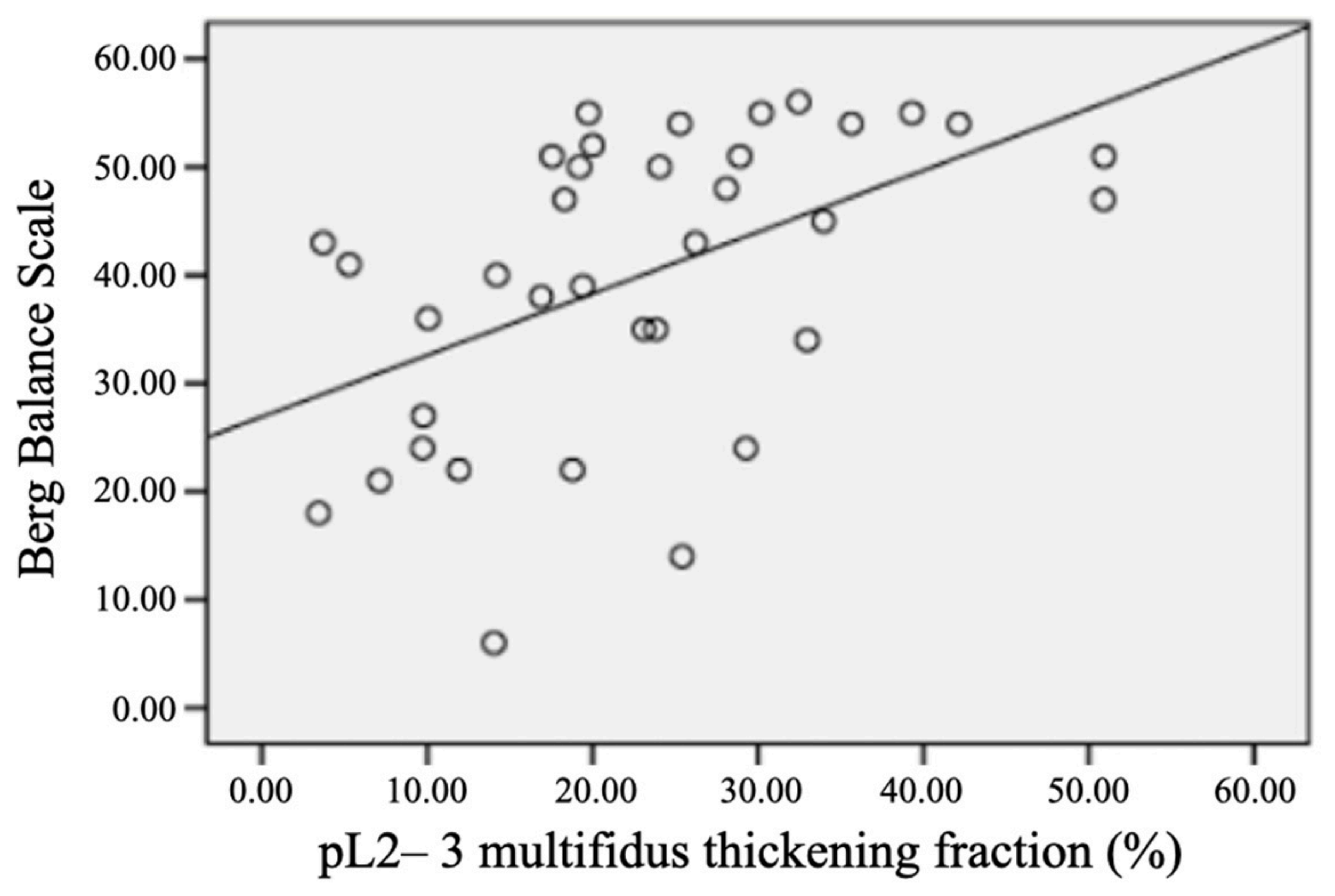
Linear regression plot illustrating the relationship between pL2–3 multifidus TF and BBS scores.

In the second block, pL2–3 multifidus TF was entered as a predictor, and the model retained statistical significance (F(3,32) = 9.04, *p* < 0.001). The addition of pL2–3 multifidus TF increased the explained variance by 10.2%, from Block 1 to a total of 40.8% (R = 0.677, R^2^ = 0.459, adjusted R^2^ = 0.408). This change was statistically significant (ΔR^2^ = 0.102, F change = 6.02, *p* = 0.020), and pL2–3 multifidus TF was a significant independent predictor of BBS (B = 0.385, 95% CI [0.065, 0.705], β = 0.340, *p* = 0.020) (Table 3). The corresponding Cohen’s f^2^ for this increment was 0.188, which is classified as a medium effect size based on standard criteria [29]. Linear regression plot illustrating the relationship between pL2–3 multifidus TF and BBS scores (Figure 3).

The multicollinearity statistics, with tolerance values ranging from 0.868 to 0.878 and VIF values of 1.14 and 1.15, indicated no significant multicollinearity in the model. Furthermore, the Durbin–Watson coefficient of 2.24 suggests that the assumption of independent residuals was satisfied.

#### 3.2.2. PASS-Total Regression Model

For the PASS-Total model, BRS–UE (r = 0.595, *p* < 0.001) was included as a control variable in Block 1 of the hierarchical regression, as it demonstrated a higher correlation with PASS-Total scores than BRS–LE (r = 0.580, *p* < 0.001). Univariate and combined regression models for BRS–UE and BRS–LE are presented in Supplementary Table S6. Furthermore, age, a well-established factor associated with balance, was included as an additional control variable in Block 1 alongside BRS–UE.

Block 1 of the hierarchical regression model, which included BRS–UE and age as control variables, explained 30.4% of the variance in PASS-Total score (R = 0.587, R^2^ = 0.344, adjusted R^2^ = 0.304, F(2,33) = 8.66, *p* < 0.001).

In the second block, TrA TFR was entered as an additional predictor; however, it did not reach statistical significance (B = 0.026, 95% CI [−0.018, 0.70], β = 0.181, *p* = 0.243). Although the model explained 31.3% of the variance in PASS-Total (R = 0.610, R^2^ = 0.372, adjusted R^2^ = 0.313), the change in R^2^ was not statistically significant (ΔR^2^ = 0.028, F change = 1.41, *p* = 0.243) (Table 4). The complete regression table, including unstandardized and standardized coefficients for all independent variables, multicollinearity diagnostics (tolerance and VIF), and autocorrelation statistics, is presented in Supplementary Table S7.

#### 3.2.3. PASS-MP Regression Model

Although BRS–UE (rs = 0.559, *p* < 0.001) showed a marginally stronger zero-order correlation with PASS-MP than BRS–LE (rs = 0.552, *p* < 0.001), its explanatory power in a single-predictor linear regression model was slightly lower, a discrepancy attributable to the rank-based nature of the Spearman coefficient versus the linear R^2^ metric (Supplementary Table S9). Consistent with the pre-specified selection strategy, BRS–UE was retained in Block 1 due to its stronger zero-order correlation with PASS-MP. Furthermore, age, a well-established factor associated with balance, was included as an additional control variable in Block 1 along with BRS–UE.

Block 1 of the hierarchical regression model, which included BRS–UE and age as control variables, explained 39.7% of the variance in PASS-MP (R = 0.657, R^2^ = 0.431, adjusted R^2^ = 0.397, F(2,33) = 12.5, *p* < 0.001).

**Figure 4:**
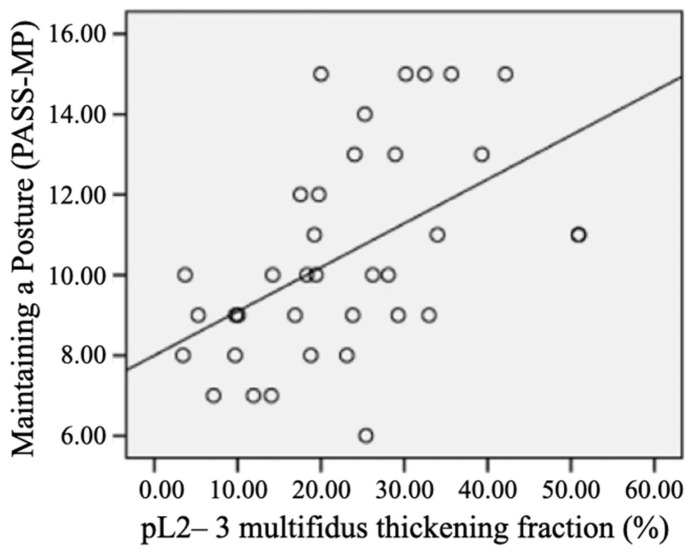
Linear regression plot illustrating the relationship between pL2–3 multifidus TF and PASS-MP scores.

In the second block, pL2–3 multifidus TF was entered as a predictor, and the model retained statistical significance (F(3,32) = 11.6, *p* < 0.001). The addition of pL2–3 multifidus TF increased the explained variance by 9%, from Block 1 to 47.7% (R = 0.722, R^2^ = 0.521, adjusted R^2^ = 0.477). This change was statistically significant (ΔR^2^ = 0.090, F change = 6.03, *p* = 0.020), and pL2–3 multifidus TF was a significant independent predictor of PASS-MP (B = 0.069, 95% CI [0.012, 0.126], β = 0.320, *p* = 0.020) (Table 4). The corresponding Cohen’s f^2^ for this increment was 0.188, which is classified as a medium effect size based on standard criteria [29]. Linear regression plot illustrating the relationship between pL2–3 multifidus TF and PASS-MP scores (Figure 4).

The multicollinearity statistics, with tolerance values ranging from 0.868 to 0.878 and VIF values of 1.14 and 1.15, indicated no significant multicollinearity in the models. Furthermore, the Durbin–Watson coefficient of 2.01 suggests that the assumption of independent residuals is satisfied.

#### 3.2.4. PASS-CP Regression Model

For the PASS-CP model, BRS–UE (r = 0.489, *p* = 0.002) was included as a control variable in Block 1 of the hierarchical regression, as it demonstrated a higher correlation with PASS-CP than BRS–LE (r = 0.428, *p* = 0.009). The univariate and combined regression models for BRS–UE and BRS–LE are presented in Supplementary Table S11. Furthermore, age, a well-established factor associated with balance, was included as an additional control variable in Block 1, alongside BRS–UE.

Block 1 of the hierarchical regression model, which included BRS–UE and age as control variables, explained 15% of the variance in PASS-CP scores (R = 0.418, R^2^ = 0.174, adjusted R^2^ = 0.150, F(1,34) = 7.18, *p* = 0.011).

In the second block, pTrA TF (B = 0.027, 95% CI [−0.020, 0.075], β = 0.189, *p* = 0.252) and TrA TFR (B = 0.009, 95% CI [−0.022, 0.040], β = 0.101, *p* = 0.558) were entered as predictors; however, neither reached statistical significance (Table 4). The complete regression tables, including unstandardized and standardized coefficients for all independent variables, multicollinearity diagnostics, and autocorrelation statistics, are presented in Supplementary Tables S12 and S13.

#### 3.2.5. Sensitivity Analysis for BRS Covariate Selection

In accordance with the Methods section, the BRS stage exhibiting the strongest zero-order (Spearman) correlation with each outcome variable was retained in Block 1 to reduce the multicollinearity. To assess the robustness of this predetermined selection strategy, sensitivity analyses were performed for outcome variables in which the zero-order correlations between BRS–UE and BRS–LE were closely aligned: BBS (r_s_ = 0.594 vs. 0.573), PASS-Total (r_s_ = 0.595 vs. 0.580), and PASS-MP (r_s_ = 0.559 vs. 0.552). For PASS-CP, the correlation difference between the two BRS stages was more distinct (r_s_ = 0.489 vs. 0.428), providing a clearer basis for covariate selection without the need for further sensitivity testing.

BBS: When BRS–LE was substituted for BRS–UE in Block 1, the pL2–3 multifidus TF continued to be a significant predictor (ΔR^2^ = 0.089, F change = 4.58, *p* = 0.040; Supplementary Table S4), mirroring the results of the original BRS–UE model (ΔR^2^ = 0.102, F change = 6.02, *p* = 0.020). A further sensitivity analysis was conducted for TrA TFR, with BRS–LE and age included in Block 1. The subsequent addition of TrA TFR in Block 2 did not achieve statistical significance (ΔR^2^ = 0.067, F change = 3.34, *p* = 0.077), consistent with its lack of significant contribution to the original BRS–UE model (Supplementary Table S5). These outcomes indicate that the significant association between pL2–3 multifidus TF and BBS and the non-significant association between TrA TFR and BBS were stable and unaffected by the choice of covariates.

PASS-Total: When BRS–LE was entered into Block 1 instead of BRS–UE, TrA TFR again did not reach statistical significance (ΔR^2^ = 0.065, F change = 3.16, *p* = 0.085), indicating that this null finding was not dependent on the selection of covariates (Supplementary Table S8).

PASS-MP: Incorporating BRS–LE instead of BRS–UE in Block 1 resulted in a smaller and statistically non-significant increase in the explained variance when the pL2–3 multifidus TF was added (ΔR^2^ = 0.061, F change = 3.79, *p* = 0.060; adjusted R^2^ = 0.438). This contrasts with the original BRS–UE model, which showed a larger and significant increase (ΔR^2^ = 0.090, F change = 6.03, *p* = 0.020; adjusted R^2^ = 0.477), as shown in the Supplementary Tables S9 and S10. When both BRS–UE and BRS–LE were simultaneously included (Supplementary Table S9, Model 3), neither variable demonstrated independent significance (β = 0.279, *p* = 0.212; β = 0.377, *p* = 0.095). Although the VIF values (VIF = 2.59) were within the conventional limits, a strong Spearman correlation was observed between the two measures (r_s_ = 0.800, *p* < 0.001), indicating substantial shared variance. This outcome indicates that, within the PASS-MP model, the statistical significance of the muscle parameter was influenced by the choice of covariate.

Taken together, these analyses indicate that BRS–UE and BRS–LE provide substantially overlapping predictive information across outcome variables. The predetermined strategy of retaining the stage with the stronger zero-order correlation did not affect the statistical conclusions for the BBS or PASS-Total. For PASS-MP, however, covariate selection did influence the statistical significance of the muscle parameter; the theoretical and empirical justifications for retaining BRS–UE in this model are elaborated in Section 4. For PASS-CP, the more pronounced difference in zero-order correlations provided sufficient a priori justification for BRS–UE selection; therefore, this outcome was not subjected to sensitivity testing.

### 3.3. Reliability Analysis of USG Measurements

The intra-rater ICC(3,1) values for all measurement points (bilateral TrA and bilateral multifidus at L2–3, L3–4, and L4–5) ranged from 0.866 to 0.927, indicating good-to-excellent reliability (Supplementary Table S14).

## 4. Discussion

The main finding of this study is that segmental activation of deep trunk muscles independently predicts functional balance and postural control in patients with hemiplegia, with detailed findings presented below. This study is a unique cross-sectional investigation in which the deep trunk muscles, TrA, and lumbar multifidus were assessed via ultrasound in patients with hemiplegia, and their predictive effects on static and dynamic balance and postural control were examined using multivariate regression analysis. The existing literature has largely focused on correlations between muscle thickness and balance measures, with regression-based studies demonstrating the relative predictive effects of independent variables being limited. Furthermore, studies that evaluate the lumbar multifidus muscle by segmenting it according to different spinal levels are rarely encountered. In this context, our study contributes by revealing the independent contribution of the segmental contraction capacity of the TrA and lumbar multifidus to balance and postural control.

### 4.1. Methodological Considerations

Certain methodological considerations must be addressed when interpreting the findings of this study. First, various clinical conditions known to affect postural control were excluded to minimize potential confounding factors that could influence the balance performance. Specifically, individuals with a history of contralateral hemiplegia, neuromuscular diseases leading to muscle atrophy, sensory deficits, cerebellar sequelae, vestibular disorders (e.g., vertigo), Parkinson’s disease, or balance impairment due to visual deficits were excluded. Patients with pre-existing balance problems prior to stroke were also excluded. These criteria aimed to reduce the influence of neurological, sensory, and musculoskeletal factors that could affect balance performance, thereby ensuring a more accurate assessment of the contribution of the deep trunk muscles to functional balance.

### 4.2. Effect of TrA and Lumbar Multifidus Activation on Balance Control

The biomechanical and neurophysiological role of the TrA in postural control is well established. By increasing intra-abdominal pressure, it enhances spinal stability and facilitates more effective control of the center of gravity [31]. Rapid TrA contraction contributes to dynamic stability by limiting trunk rotations, whereas involuntary pre-movement contractions—via a feedforward mechanism—stabilize the trunk before limb movement begins, preventing loss of balance [32,33,34,35]. In this study, all paretic-side TrA parameters, except resting thickness, were lower than those on the non-paretic side. This finding is consistent with previous studies reporting reduced TrA contraction ratios on the paretic side compared to the unaffected side [36]. Furthermore, while pTrA TF correlated only with PASS-CP, TrA TFR correlated with all dependent variables except PASS-MP. Contrary to expectations, neither parameter demonstrated a statistically significant predictive effect in the regression analyses. Measurements were taken following the ADIM, as Goldman et al. demonstrated in an EMG study that this maneuver selectively activates the TrA muscle [37]. In line with our findings, Kołcz et al. reported that TrA activity is unrelated to balance. Similarly, they observed that neither the patient nor the healthy group were able to contract their muscles effectively in a controlled and conscious manner [38]. The patients in our study also had considerable difficulty performing the ADIM correctly, which may have resulted in inadequate TrA activation during measurement, potentially explaining why TrA parameters failed to reach statistical significance in the regression models. Additionally, the mean resting thickness ratio exceeding 100% in our sample may reflect increased resting muscle tone in the trunk, consistent with the spasticity observed in the extremities, potentially attenuating the predictive effect of TrA parameters.

TSEs improve functional mobility, walking speed, and postural control in patients with hemiplegia by increasing TrA thickness. When combined with conventional therapy, TSE significantly improves PASS and Barthel Index scores [39,40,41]. These findings are consistent with the correlations observed in our study between TrA parameters and PASS-Total and PASS-CP scores; however, there is no definitive evidence that this improvement is directly attributable to TrA activation alone. Although significant improvements were observed in both muscle thickness and balance measures following exercise, this does not establish that changes in balance outcomes were solely driven by changes in muscle thickness.

The deep lumbar multifidus muscle plays a critical role in lumbar stabilization and directly contributes to dynamic sagittal imbalance through abnormal activation and fatigue [42]. The likelihood of effectively contracting the multifidus muscle is significantly increased in patients who can effectively contract the TrA, and the contraction capacities of these two muscles are strongly correlated [43]. Both muscles contract early via a feedforward mechanism prior to lower extremity movements to control trunk flexion and maintain the center of mass over the support surface [34]. Collectively, these findings demonstrate that TrA thickness measurements alone are insufficient to capture its functional contribution to balance and that activation pattern and timing may play a critical role in postural control.

In this study, the lumbar multifidus muscle activation was assessed in three spinal segments. Although all paretic-side multifidus parameters were lower than those on the non-paretic side, statistically significant differences in contractility were observed only in the L4–5 segment. In the correlation analyses, pL2–3 multifidus TF demonstrated a significant association with BBS and PASS-MP, whereas no significant correlations were found between the other multifidus parameters and the dependent variables. In the regression models, pL2–3 multifidus TF was identified as an independent predictor of BBS and PASS-MP. These findings suggest that upper lumbar multifidus segments may be more closely associated with trunk stability, whereas lower lumbar segments may be more relevant to pelvic stability.

The medium effect size (f^2^ = 0.188 for both BBS and PASS-MP models) linked to pL2–3 multifidus TF implies that, although it cannot independently determine balance performance, it may still offer a valuable and clinically significant addition to existing assessment tools, such as BRS–UE. In the context of rehabilitation, evaluating the function of deep trunk muscles, such as the multifidus, could enhance the information provided by motor staging scales. Nevertheless, this parameter should be viewed as part of a broader, multidimensional evaluation rather than as the sole basis for clinical decisions.

As detailed in the Methods section, a modified maximal trunk extension maneuver was used instead of the standard contralateral arm/leg lift protocol. This decision was necessary because of the severe lower extremity motor deficits and physical inability to perform upper extremity movements in patients with BRS-UE stage 1, which rendered the standard protocol impractical. Although this maneuver relies on the function of the multifidus as a primary lumbar extensor [27], it assumes that maximal trunk extension engages the multifidus similarly to the standard contralateral limb raise task. This assumption has not been directly validated against the conventional protocol, and the direction and magnitude of any differences in the resulting thickening fraction values remain unknown. Ultimately, thickening fraction values obtained with the modified maneuver should be interpreted with this methodological distinction in mind, rather than being directly equivalent to those reported using the standard task.

Conflicting findings in the literature largely stem from methodological differences. While studies conducted in the acute phase of stroke report significant correlations between trunk muscle contraction ratios and balance, chronic-phase studies relying solely on resting muscle thickness often find no association with balance and no morphological differences between sides, possibly because static thickness measurements do not adequately capture dynamic muscle function [4,44].

Suh et al. identified posterior trunk muscle thickness as a predictor of balance and ambulation in patients with subacute stroke; however, significant contributions from abdominal muscles could not be demonstrated, likely because static thickness measurements do not adequately reflect dynamic muscle function [45]. Studies comparing TSE and neuromuscular electrical stimulation (NMES) for trunk muscles have demonstrated balance improvements with both approaches. Combination therapy yielded greater gains in BBS compared to NMES alone, while NMES applied to both abdominal and posterior trunk muscles similarly improved balance, supporting the notion that all deep trunk muscles contribute independently to postural control [46,47].

The motor recovery stage (BRS) was also a significant predictor of balance and postural outcomes. The BRS–LE stage independently predicted BBS, PASS-Total, and PASS-MP, whereas the BRS–UE stage independently predicted all dependent variables. Because BRS–UE and BRS–LE were highly correlated (rs = 0.800, *p* < 0.001), the BRS–UE stage likely masked the effect of BRS–LE in the regression analyses; together with the pre-specified statistical criteria outlined in Section 3.2.5, this informed our decision to retain BRS–UE as the covariate in Block 1, including for the PASS-MP model, where the zero-order correlations between the two BRS stages were closely aligned. This choice is consistent with existing evidence on the role of upper-limb motor function in balance and postural control after stroke: Arya et al. reported a significant association between upper- and lower-extremity motor function and BBS and PASS-Total in chronic-phase stroke [48], arm movements occur before and during balance perturbations and contribute to balance recovery [49,50], and balance and mobility improve when arm movement is unrestricted [51]. These findings support BRS–UE as a stronger and theoretically more relevant predictor in our study.

Sensitivity analyses substituting BRS–LE for BRS–UE showed consistent results for BBS and PASS-Total, whose associations with pL2–3 multifidus TF and TrA TFR, respectively, were unaffected by the choice of covariates. PASS-MP was the only outcome affected: substituting BRS–LE markedly reduced the explanatory power of pL2–3 multifidus TF, reflecting the substantial shared variance between BRS–UE and BRS–LE (rs = 0.800, r^2^ = 0.64). Although the VIF remained within conventional limits (VIF = 2.59), such thresholds may underestimate coefficient instability in small samples with this degree of shared variance. Overall, the association between pL2–3 multifidus TF and PASS-MP appears substantial rather than an artifact of covariate selection, persisting after accounting for upper-limb motor recovery and corroborated by consistent trends across other outcomes.

Furthermore, a regression-based study consistent with our findings reported no significant effect of age—the other control variable in our study—on balance. [26].

### 4.3. Strengths and Limitations

This study has several significant strengths. The segmental ultrasound evaluation of the transversus abdominis (TrA) and lumbar multifidus muscles, along with their independent contributions to balance assessed through hierarchical regression, represents a methodological approach that has been explored in only a limited number of studies. The high intra-rater reliability of the ultrasonographic measurements, combined with the strong explanatory power and significant effect sizes observed in the regression models, provides compelling evidence of a relationship between the activation of segmental deep trunk muscles and post-stroke balance and postural control.

Given that the sample was derived from a single center and consisted of a relatively small, selected patient cohort, the statistical power and generalizability of the findings to the broader stroke population may be constrained. The cross-sectional nature of the study precluded the establishment of causal relationships between trunk muscle parameters and balance outcomes. A negative correlation was identified between age and most dependent variables (excluding PASS-CP); however, the relatively narrow age range may have diminished this variable’s contribution in multivariate models. The sample comprised both subacute (<6 months, *n* = 20) and chronic (≥6 months, *n* = 16) patients, with a mean time since onset of 17.64 ± 23.14 months. The limited sample size precluded formal adjustment for time since stroke or subgroup comparison, although an exploratory analysis revealed no significant correlation between time since onset and any primary balance outcome (BBS: r_s_ = −0.101, *p* = 0.558; PASS-Total: r_s_ = −0.016, *p* = 0.928; PASS-MP: r_s_ = −0.115, *p* = 0.504; PASS-CP: r_s_ = 0.073, *p* = 0.673). This provides preliminary reassurance against confounding by chronicity, although it does not substitute for a formally powered subgroup analysis. Future studies with larger, chronicity-stratified samples are necessary to confirm consistency across recovery stages. Furthermore, the absence of a healthy control group meant that physiological side-to-side asymmetry in age-matched individuals was not quantified, and the potential influence of neurological lateralization or hand/limb dominance on the observed asymmetries could not be assessed. Future research should incorporate age-matched healthy controls and evaluate both dominance and stroke laterality to differentiate stroke-related asymmetry from physiological variation.

The residuals from Block 1 of the hierarchical regression models exhibited a minor deviation from normality on the left tail, which should be considered when interpreting the results. Given that ultrasonographic thickness represents the structural or mechanical aspects of muscle activation rather than direct neuromuscular timing, motor unit recruitment, or feedforward postural control, and considering that trunk muscle spasticity cannot be quantitatively assessed, future research should incorporate electromyography or other neuromuscular assessment methods. Among the 60 exploratory correlations examined without adjustment for multiple comparisons, a subsequent Bonferroni correction confirmed that the primary finding, that is, the association between the pL2–3 multifidus thickening fraction and balance performance, remained valid, whereas several other correlations did not withstand correction and should be considered hypothesis-generating. Lastly, the use of a modified maximal trunk extension maneuver instead of the standard contralateral arm/leg lift protocol limits the direct comparability of thickening fraction values with previous studies using the conventional protocol; therefore, standardized activation protocols tailored to stroke populations are necessary.

## 5. Conclusions

This study demonstrates that the functional status of deep trunk muscles in patients with post-stroke hemiplegia is associated with balance and postural control. The findings suggest that the activation capacity of the paretic-side lumbar multifidus, particularly at the upper lumbar segment, is associated with functional balance, while TrA activation may also be associated with the maintenance of postural control. These results indicate that trunk stability in stroke may be related to not only limb motor function but also the segmental activation characteristics of deep trunk muscles.

From a translational perspective, these findings have important implications for the clinical planning of trunk stabilization interventions in stroke rehabilitation. Individualized, task-oriented core stabilization exercises targeting segmental lumbar multifidus and TrA activation during early rehabilitation may improve balance and postural control in patients with stroke. The integration of ultrasonographic imaging into clinical practice may enable real-time monitoring of muscle function and facilitate tailoring of treatment programs to individual patient needs.

Future studies with larger samples and long-term follow-ups are needed to clarify the effects of deep trunk muscle-targeted rehabilitation on balance, functional mobility, and activities of daily living.

## Figures and Tables

**Table 1:** TrA and lumbar multifidus muscle thickness measurements and muscle ratios.

	Paretic-Side	Non-Paretic Side	*p*
	(Mean ± SD)	(Mean ± SD)	
Transversus abdominis			
Resting thickness (mm)	3.38 ± 0.81	3.46 ± 0.81	0.685
Contraction thickness (mm)	5.23 ± 1.25	6.14 ± 1.46	0.008
Thickening fraction (%)	56.12 ± 22.15	79.4 ± 29.71	0.006
L2–3 multifidus			
Resting thickness (mm)	30.56 ± 6.15	31.56 ± 6.36	0.512
Contraction thickness (mm)	37.27 ± 6.7	39.46 ± 6.5	0.176
Thickening fraction (%)	23 ± 12.34	26.61 ± 14.78	0.278
L3–4 multifidus			
Resting thickness (mm)	32.6 ± 6.24	33.6 ± 6.68	0.526
Contraction thickness (mm)	40.14 ± 6.36	42.15 ± 6.87	0.215
Thickening fraction (%)	24.43 ± 12.64	26.95 ± 14.25	0.443
L4–5 multifidus	32.48 ± 6.04	32.6 ± 5.98	0.935
Resting thickness (mm)	39.11 ± 5.95	41.29 ± 5.89	0.134
Contraction thickness (mm)	21.59 ± 11.5	28.25 ± 14.35	0.039
Thickening fraction (%)			
	Mean ± SD		
Transversus abdominis			
Resting thickness ratio (%)	100.37 ± 21.18		
Thickening fraction ratio (%)	76.16 ± 34.85		
L2–3 multifidus			
Resting thickness ratio (%)	97.20 ± 8.33		
Thickening fraction ratio (%)	91.11 ± 28.55		
L3–4 multifidus			
Resting thickness ratio (%)	97.63 ± 8.18		
Thickening fraction ratio (%)	91.23 ± 26.20		
L4–5 multifidus			
Resting thickness ratio (%)	99.96 ± 8.31		
Thickening fraction ratio (%)	82.03 ± 31.49		

SD: Standard Deviation; L2–3, L3–4, L4–5: Lumbar vertebral levels.

**Table 2:** Correlation analysis between independent and dependent variables.

	BBS		PASS-Total		PASS-MP		PASS-CP	
	r	*p*	r	*p*	r	*p*	r	*p*
BRS								
Upper extremity stage	**0.594**	**<0.001**	**0.595**	**<0.001**	**0.559**	**<0.001**	**0.489**	**0.002**
Lower extremity stage	**0.573**	**<0.001**	**0.580**	**<0.001**	**0.552**	**<0.001**	**0.428**	**0.009**
Age	**−0.467**	**0.004**	**−0.380**	**0.022**	**−0.513**	**0.001**	−0.255	0.133
Transversus Abdominis								
Paretic-side muscle TF (%)	0.241	0.157	0.321	0.056	0.229	0.180	**0.347**	**0.038**
Resting thickness ratio (%)	−0.213	0.211	−0.149	0.385	−0.179	0.297	−0.109	0.526
TF ratio (%)	**0.376**	**0.024**	**0.370**	**0.027**	0.308	0.068	**0.379**	**0.022**
L2–3 Multifidus								
Paretic-side muscle TF (%)	**0.553**	**<0.001**	0.325	0.053	**0.583**	**<0.001**	0.274	0.105
Resting thickness ratio (%)	0.085	0.624	0.123	0.475	0.083	0.631	0.123	0.476
TF ratio (%)	0.116	0.500	0.209	0.222	0.107	0.535	0.181	0.291
L3–4 Multifidus								
Paretic-side muscle TF (%)	0.317	0.060	0.185	0.281	0.292	0.084	0.060	0.728
Resting thickness ratio (%)	0.199	0.245	0.227	0.184	0.113	0.513	0.162	0.345
TF ratio (%)	−0.013	0.939	0.029	0.866	−0.051	0.768	0.052	0.761
L4–5 Multifidus								
Paretic-side muscle TF (%)	0.096	0.579	−0.006	0.971	0.102	0.556	−0.055	0.751
Resting thickness ratio (%)	0.018	0.919	0.007	0.968	−0.066	0.700	0.037	0.831
TF ratio (%)	−0.213	0.213	−0.261	0.124	−0.217	0.203	−0.211	0.217

BBS: Berg Balance Scale; PASS: Postural Assessment Scale for Stroke Patients; PASS-MP: Maintaining a Posture Subscale; PASS-CP: Changing a Posture Subscale; BRS: Brunnstrom Stage of Recovery; TF: Thickening Fraction; L2–3, L3–4, L4–5: Lumbar vertebral levels. Bold text indicates statistically significant *p*-values (*p* < 0.05).

**Table 3:** Hierarchical Regression Models for BBS.

Model	R	R^2^	Adjusted R^2^	F	df1	df2	*p*
1	0.597	0.357	0.318	9.16	2	33	<0.001
2	0.677	0.459	0.408	9.04	3	32	<0.001
	ΔR^2^ = 0.102			F change = 6.02, *p* = 0.020			
Model Coefficients							
			95% CI				
Predictor	B	SE	Lower	Upper	Beta	t	*p*
(Constant)	43.806	22.236	−1.487	89.099		1.97	0.058
BRS–UE	3.548	1.257	0.989	6.108	0.394	2.82	0.008
Age	−0.454	0.345	−1.157	0.248	−0.184	−1.32	0.197
pL2–3 multifidus TF	0.385	0.157	0.065	0.705	0.340	2.45	0.020

CI: Confidence Interval; BRS–UE: Brunnstrom Stage of Recovery of the Upper Extremity; *p*: Paretic-side; L2–3: Lumbar vertebral level; TF: Thickening Fraction.

**Table 4:** Hierarchical Regression Models for PASS-Total and Its Subscales.

**PASS-Total Regression Model**								
	**Model**	**R**	**R^2^**	**Adjusted R^2^**	**F**	**df1**	**df2**	***p***
	1	0.587	0.344	0.304	8.66	2	33	<0.001
	2	0.610	0.372	0.313	6.32	3	32	0.002
		ΔR^2^ = 0.028			F change = 1.41, *p* = 0.243			
**PASS-MP Regression Model**								
	**Model**	**R**	**R^2^**	**Adjusted R^2^**	**F**	**df1**	**df2**	***p***
	1	0.657	0.431	0.397	12.5	2	33	<0.001
	2	0.722	0.521	0.477	11.6	3	32	<0.001
		ΔR^2^ = 0.090			F change = 6.03, *p* = 0.020			
Model Coefficients—PASS-MP								
				95% CI				
Predictor		B	SE	Lower	Upper	Beta	t	*p*
(Constant)		13.446	3.979	5.341	21.551		3.38	0.002
BRS–UE		0.694	0.225	0.236	1.152	0.405	3.09	0.004
Age		−0.125	0.062	−0.250	0.001	−0.264	−2.02	0.052
pL2–3 multifidus TF		0.069	0.028	0.012	0.126	0.320	2.46	0.020
**PASS-CP Regression Model**								
	**Model**	**R**	**R^2^**	**Adjusted R^2^**	**F**	**df1**	**df2**	***p***
	1	0.418	0.174	0.150	7.18	1	34	0.011
	2	0.455	0.207	0.159	4.31	2	33	0.022
	3	0.428	0.183	0.134	3.70	2	33	0.036
		(Model 1–2) ΔR^2^ = 0.033			F change = 1.36, *p* = 0.252			
		(Model 1–3) ΔR^2^ = 0.009			F change = 0.35, *p* = 0.558			

PASS: Postural Assessment Scale for Stroke Patients; PASS-MP: Maintaining a Posture Subscale; CI: Confidence Interval; BRS–UE: Brunnstrom Stage of Recovery of the Upper Extremity; TF: Thickening Fraction; L2–3: Lumbar vertebral level; PASS-CP: Changing a Posture Subscale.

## Data Availability

The data presented in this study are available on request from the corresponding author. The data are not publicly available due to privacy and ethical restrictions.

## Supplementary Materials

The following supporting information can be downloaded at: https://www.mdpi.com/article/10.3390/jcm15155763/s1. Table S1. Correlation analysis of dependent variables and BRS stages. Table S2. BBS Regression Models (BRS–UE and BRS–LE). Table S3. BBS Regression Model (BRS–UE, Age, and TrA TFR). Table S4. BBS Regression Model (BRS–LE, Age, and pL2–3 multifidus TF). Table S5. BBS Regression Model (BRS–LE, Age, and TrA TFR). Table S6. PASS-Total Regression Models (BRS–UE and BRS–LE). Table S7. PASS-Total Regression Model (BRS-UE, Age, and TrA TFR). Table S8. PASS-Total Regression Model (BRS-LE, Age, and TrA TFR). Table S9. PASS-MP Regression Models (BRS–UE and BRS–LE). Table S10. PASS-MP Regression Model (BRS–LE, Age, and pL2–3 multifidus TF). Table S11. PASS-CP Regression Model (BRS–UE and BRS–LE). Table S12. PASS-CP Regression Model (BRS–UE, Age, and pTrA TF). Table S13. PASS-CP Regression Model (BRS–UE, Age, and TrA TFR). Table S14. Reliability Analysis of USG Measurements.

## Abbreviations

The following abbreviations are used in this manuscript:

ADIM	Abdominal drawing-in maneuver
ADLs	Activities of daily living
BBS	Berg Balance Scale
BRS	Brunnstrom Stage of Recovery
CI	Confidence interval
CP	Changing a posture
DALYs	Disability-adjusted life-years
ICC	Intraclass correlation coefficient
LE	Lower extremity
MP	Maintaining a posture
PASS	Postural Assessment Scale for Stroke Patients
pL2–3	Paretic-side L2–3
pTrA	Paretic-side Tranversus Abdominis
RTR	Resting thickness ratio
TF	Thickening fraction
TFR	Thickening fraction ratio
TrA	Transversus Abdominis
TSE	Trunk stabilization exercise
UE	Upper extremity
USG	Ultrasonography
VIF	Variance Inflation Factor
