# Supplementary material for: The Effect of Lumbar Multifidus and Transversus Abdominis Muscle Activation on Balance in Patients with Hemiplegia
Source: J Clin Med. 2026 Jul 23;15(15):5763. doi: 10.3390/jcm15155763 (PMC13467171; doi:10.3390/jcm15155763)
Supplement: Supplementary file 1 [file jcm-15-05763-s001.zip › jcm-4386715-supplementary.pdf]

Supplementary Materials

Supplementary Table S1. Correlation analysis of dependent variables and BRS stages.

		BBS	PASS-Total	PASS-MP	PASS-CP	BRS–UE	BRS–LE
**BBS**	r_s	—					
	p	—					
**PASS-Total**	r_s	0.917	—				
	p	<0.001	—				
**PASS-MP**	r_s	0.955	0.921	—			
	p	<0.001	<0.001	—			
**PASS-CP**	r_s	0.789	0.887	0.734	—		
	p	<0.001	<0.001	<0.001	—		
**BRS–UE**	r_s	0.594	0.595	0.559	0.489	—	
	p	<0.001	<0.001	<0.001	0.002	—	
**BRS–LE**	r_s	0.573	0.580	0.552	0.428	0.800	—
	p	<0.001	<0.001	<0.001	0.009	<0.001	—

BBS: Berg Balance Scale; PASS: Postural Assessment Scale for Stroke Patients; PASS-MP: Maintaining a Posture Subscale; PASS-CP: Changing a Posture Subscale; BRS–UE: Brunnstrom Stage of Recovery of the Upper Extremity; BRS–LE: Brunnstrom Stage of Recovery of the Lower Extremity; r_s: Spearman's rank correlation coefficient.

Supplementary Table S2. BBS Regression Models (BRS–UE and BRS–LE)

						BRS–UE		BRS–LE	
**Model**	**R**	**R²**	**Adjusted R²**	**F**	**p**	**Beta**	**p**	**Beta**	**p**
1	0.545	0.297	0.276	14.4	<0.001	0.545	<0.001		
2	0.485	0.235	0.213	10.5	0.003			0.485	0.003
3	0.553	0.306	0.264	7.27	0.002	0.428	0.076	0.150	0.525
Multicollinearity and autocorrelation statistics									
**Tolerance**			**VIF**			**Durbin-Watson**			
0.387			2.59			2.06			

BRS–UE: Brunnstrom Stage of Recovery of the Upper Extremity; BRS–LE: Brunnstrom Stage of Recovery of the Lower Extremity; VIF: Variance Inflation Factor.

Supplementary Table S3. BBS Regression Model (BRS–UE, Age, and TrA TFR)

Model	R	R²	Adjusted R²	F	df1	df2	p
1	0.597	0.357	0.318	9.16	2	33	<0.001
2	0.623	0.388	0.331	6.77	3	32	0.001
	$\Delta R^2 = 0.037$			F change = 1.83, p = 0.185			

Model Coefficients							
			95% CI				
**Predictor**	**B**	**SE**	**Lower**	**Upper**	**Beta**	**t**	**p**
(Constant)	55.297	22.873	8.705	101.888		2.42	0.021
BRS–UE	3.591	1.394	0.751	6.431	0.399	2.58	0.015
Age	-0.605	0.359	-1.336	0.127	-0.244	-1.68	0.102
TrA TFR	0.075	0.059	-0.045	0.195	0.192	1.28	0.210

Multicollinearity and autocorrelation statistics				
**Model**	**Predictor**	**Tolerance**	**VIF**	**DW**
1	BRS–UE and Age	0.912	1.10	2.18
2	BRS–UE<br>Age<br>TrA TFR	0.797<br>0.908<br>0.849	1.25<br>1.10<br>1.18	2.26

CI: Confidence Interval; BRS–UE: Brunnstrom Stage of Recovery of the Upper Extremity; TrA: Transversus Abdominis; TFR: Thickening Fraction Ratio; VIF: Variance Inflation Factor; DW: Durbin-Watson.

Supplementary Table S4. BBS Regression Model (BRS–LE, Age, and pL2-3 multifidus TF)

**Model**	**R**	**R^2**	**Adjusted R^2**	**F**	**df1**	**df2**	**p**
1	0.535	0.286	0.243	6.61	2	33	0.004
2	0.613	0.375	0.317	6.41	3	32	0.002
	$\Delta R^2 = 0.089$			F change = 4.58, p = 0.040			

Model Coefficients							
			95% CI				
**Predictor**	**B**	**SE**	**Lower**	**Upper**	**Beta**	**t**	**p**
(Constant)	47.615	25.386	-4.095	99.325		1.88	0.070
BRS–LE	2.992	1.842	-0.761	6.745	0.264	1.62	0.114
Age	-0.496	0.379	-1.267	0.275	-0.200	-1.31	0.199
pL2-3 multifidus TF	0.378	0.177	0.018	0.738	0.334	2.14	0.040

Multicollinearity and autocorrelation statistics				
**Model**	**Predictor**	**Tolerance**	**VIF**	**DW**
1	BRS–LE and Age	0.851	1.18	2.39
2	BRS–LE<br>Age<br>pL2-3 multifidus TF	0.738<br>0.835<br>0.801	1.35<br>1.20<br>1.25	2.42

CI: Confidence Interval; BRS–LE: Brunnstrom Stage of Recovery of the Lower Extremity; p: paretic-side; TF: Thickening Fraction; VIF: Variance Inflation Factor; DW: Durbin-Watson.

Supplementary Table S5. BBS Regression Model (BRS–LE, Age, and TrA TFR)

Model	R	R^2	Adjusted R^2	F	df1	df2	p
1	0.535	0.286	0.243	6.61	2	33	0.004
2	0.595	0.353	0.293	5.83	3	32	0.003
	$\Delta R^2 = 0.067$			F change = 3.34, p = 0.077			

Model Coefficients

Predictor	B	SE	95% CI Lower	95% CI Upper	Beta	t	p
(Constant)	47.722	25.934	-5.103	100.547		1.84	0.075
BRS–LE	3.801	1.779	0.176	7.425	0.335	2.14	0.040
Age	-0.544	0.383	-1.325	0.236	-0.220	-1.42	0.165
TrA TFR	0.105	0.058	-0.012	0.223	0.269	1.83	0.077

Multicollinearity and autocorrelation statistics

Model	Predictor	Tolerance	VIF	DW
1	BRS–LE and Age	0.851	1.18	2.39
2	BRS–LE<br>Age<br>TrA TFR	0.819<br>0.844<br>0.935	1.22<br>1.18<br>1.07	2.46

CI: Confidence Interval; BRS–LE: Brunnstrom Stage of Recovery of the Lower Extremity; TrA: Transversus Abdominis; TFR: Thickening Fraction Ratio; VIF: Variance Inflation Factor; DW: Durbin-Watson.

Supplementary Table S6. PASS-Total Regression Models (BRS–UE and BRS–LE)

						BRS–UE		BRS–LE	
Model	R	R^2	Adjusted R^2	F	p	Beta	p	Beta	p
1	0.566	0.320	0.300	16.0	<0.001	0.566	<0.001		
2	0.509	0.259	0.237	11.9	0.002			0.509	0.002
3	0.575	0.331	0.291	8.17	0.001	0.432	0.068	0.170	0.463

Multicollinearity and autocorrelation statistics

Tolerance	VIF	Durbin-Watson
0.387	2.59	2.43

BRS–UE: Brunnstrom Stage of Recovery of the Upper Extremity; BRS–LE: Brunnstrom Stage of Recovery of the Lower Extremity; VIF: Variance Inflation Factor.

Supplementary Table S7. PASS-Total Regression Model (BRS-UE, Age, and TrA TFR)

Model	R	R^2	Adjusted R^2	F	df1	df2	p
1	0.587	0.344	0.304	8.66	2	33	<0.001
2	0.610	0.372	0.313	6.32	3	32	0.002
	$\Delta R^2 = 0.028$			F change = 1.41, p = 0.243			

Model Coefficients							
			95% CI				
**Predictor**	**B**	**SE**	**Lower**	**Upper**	**Beta**	**t**	**p**
(Constant)	30.466	8.425	13.304	47.627		3.62	0.001
BRS–UE	1.477	0.513	0.431	2.523	0.451	2.88	0.007
Age	-0.136	0.132	-0.406	0.133	-0.152	-1.03	0.310
TrA TFR	0.026	0.022	-0.018	0.070	0.181	1.19	0.243

Multicollinearity and autocorrelation statistics				
**Model**	**Predictor**	**Tolerance**	**VIF**	**DW**
1	BRS–UE and Age	0.912	1.10	2.47
2	BRS–UE<br>Age<br>TrA TFR	0.797<br>0.908<br>0.849	1.25<br>1.10<br>1.18	2.60

CI: Confidence Interval; BRS–UE: Brunnstrom Stage of Recovery of the Upper Extremity; TrA: Transversus Abdominis; TFR: Thickening Fraction Ratio; VIF: Variance Inflation Factor; DW: Durbin-Watson.

Supplementary Table S8. PASS-Total Regression Model (BRS-LE, Age, and TrA TFR)

**Model**	**R**	**R^2**	**Adjusted R^2**	**F**	**df1**	**df2**	**p**
1	0.525	0.276	0.232	6.28	2	33	0.005
2	0.584	0.341	0.279	5.52	3	32	0.004
	$\Delta R^2 = 0.065$			F change = 3.16, p = 0.085			

Model Coefficients							
			95% CI				
**Predictor**	**B**	**SE**	**Lower**	**Upper**	**Beta**	**t**	**p**
(Constant)	26.662	9.520	7.271	46.053		2.80	0.009
BRS–LE	1.649	0.653	0.319	2.980	0.400	2.525	0.017
Age	-0.010	0.141	-0.391	0.181	-0.117	-0.747	0.461
TrA TFR	0.038	0.021	-0.005	0.081	0.264	1.778	0.085

Multicollinearity and autocorrelation statistics				
**Model**	**Predictor**	**Tolerance**	**VIF**	**DW**
1	BRS–LE and Age	0.851	1.18	2.71
2	BRS–LE<br>Age<br>TrA TFR	0.819<br>0.844<br>0.935	1.22<br>1.18<br>1.07	2.87

CI: Confidence Interval; BRS–LE: Brunnstrom Stage of Recovery of the Lower Extremity; TrA: Transversus Abdominis; TFR: Thickening Fraction Ratio; VIF: Variance Inflation Factor; DW: Durbin-Watson.

Supplementary Table S9. PASS-MP Regression Models (BRS–UE and BRS–LE)

						BRS–UE		BRS–LE	
**Model**	**R**	**R^2**	**Adjusted R^2**	**F**	**p**	**Beta**	**p**	**Beta**	**p**
1	0.575	0.330	0.311	16.8	<0.001	0.575	<0.001		
2	0.596	0.355	0.336	16.7	<0.001			0.596	<0.001
3	0.621	0.385	0.348	10.3	<0.001	0.279	0.212	0.377	0.095

Multicollinearity and autocorrelation statistics

Tolerance	VIF	Durbin-Watson
0.387	2.59	2.00

BRS–UE: Brunnstrom Stage of Recovery of the Upper Extremity; BRS–LE: Brunnstrom Stage of Recovery of the Lower Extremity; VIF: Variance Inflation Factor.

Supplementary Table S10. PASS-MP Regression Model (BRS–LE, Age, and pL2-3 multifidus TF)

Model	R	R^2	Adjusted R^2	F	df1	df2	p
1	0.652	0.425	0.390	12.2	2	33	<0.001
2	0.697	0.486	0.438	10.1	3	32	<0.001
	$\Delta R^2 = 0.061$			F change = 3.79, p = 0.060			

Model Coefficients

			95% CI				
**Predictor**	**B**	**SE**	**Lower**	**Upper**	**Beta**	**t**	**p**
(Constant)	12.577	4.383	3.649	21.504		2.87	0.007
BRS–LE	0.821	0.318	0.173	1.469	0.381	2.58	0.015
Age	-0.118	0.065	-0.251	0.015	-0.250	-1.80	0.081
pL2-3 multifidus TF	0.059	0.030	-0.003	0.121	0.276	1.95	0.060

Multicollinearity and autocorrelation statistics

Model	Predictor	Tolerance	VIF	DW
1	BRS–LE and Age	1.000	1.00	–
2	BRS–LE	0.738	1.35	2.26
	Age	0.835	1.20	
	pL2-3 multifidus TF	0.801	1.25	

CI: Confidence Interval; BRS–LE: Brunnstrom Stage of Recovery of the Lower Extremity; p: Paretic-side; L2–3: Lumbar vertebral level; TF: Thickening Fraction; VIF: Variance Inflation Factor; DW: Durbin-Watson.

Supplementary Table S11. PASS-CP Regression Model (BRS–UE and BRS–LE)

						BRS–UE		BRS–LE	
**Model**	**R**	**R^2**	**Adjusted R^2**	**F**	**p**	**Beta**	**p**	**Beta**	**p**
1	0.418	0.174	0.150	7.18	0.011	0.418	0.011		
2	0.310	0.096	0.070	3.62	0.065			0.310	0.065
3	0.418	0.175	0.125	3.50	0.042	0.451	0.085	-0.043	0.866

Multicollinearity and autocorrelation statistics		
Tolerance	VIF	Durbin-Watson
0.387	2.59	2.42

BRS–UE: Brunnstrom Stage of Recovery of the Upper Extremity; BRS–LE: Brunnstrom Stage of Recovery of the Lower Extremity; VIF: Variance Inflation Factor.

Supplementary Table S12. PASS-CP Regression Model (BRS–UE, Age, and pTrA TF)

Model	R	R^2	Adjusted R^2	F	df1	df2	p
1	0.418	0.175	0.125	3.49	2	33	0.042
2	0.455	0.207	0.133	2.79	3	32	0.057
	$\Delta R^2 = 0.032$			F change = 1.31, p = 0.261			

Model Coefficients							
			95% CI				
Predictor	B	SE	Lower	Upper	Beta	t	p
(Constant)	15.501	5.919	3.445	27.558		2.619	0.013
BRS–UE	0.750	0.360	0.017	1.483	0.361	2.084	0.045
Age	-0.003	0.095	-0.196	0.190	-0.006	-0.035	0.972
pTrA TF	0.027	0.024	-0.021	0.076	0.190	1.145	0.261

Multicollinearity and autocorrelation statistics				
Model	Predictor	Tolerance	VIF	DW
1	BRS–UE and Age	0.912	1.10	2.43
2	BRS–UE	0.824	1.21	2.54
	Age	0.898	1.11	
	pTrA TF	0.903	1.11	

CI: Confidence Interval; BRS–UE: Brunnstrom Stage of Recovery of the Upper Extremity; p: Paretic-side; TrA: Transversus Abdominis; TF: Thickening Fraction; VIF: Variance Inflation Factor; DW: Durbin-Watson.

Supplementary Table S13. PASS-CP Regression Model (BRS–UE, Age, and TrA TFR)

Model	R	R^2	Adjusted R^2	F	df1	df2	p
1	0.418	0.175	0.125	3.49	2	33	0.042
2	0.428	0.184	0.107	2.40	3	32	0.086
	$\Delta R^2 = 0.009$			F change = 0.35, p = 0.558			

Model Coefficients							
			95% CI				
Predictor	*B*	*SE*	Lower	Upper	Beta	t	p
(Constant)	15.165	6.093	2.755	27.575		2.489	0.018
BRS–UE	0.800	0.371	0.044	1.557	0.385	2.155	0.039
Age	0.014	0.096	-0.181	0.209	0.024	0.145	0.886
TrA TFR	0.009	0.016	-0.023	0.041	0.102	0.591	0.558

Multicollinearity and autocorrelation statistics				
**Model**	**Predictor**	**Tolerance**	**VIF**	**DW**
1	BRS–UE and Age	0.912	1.10	2.43
2	BRS–UE<br>Age<br>TrA TFR	0.797<br>0.908<br>0.849	1.25<br>1.10<br>1.18	2.48

CI: Confidence Interval; BRS–UE: Brunnstrom Stage of Recovery of the Upper Extremity; TrA: Transversus Abdominis; TFR: Thickening Fraction Ratio; VIF: Variance Inflation Factor; DW: Durbin-Watson.

Supplementary Table S14. Reliability Analysis of USG Measurements

**Muscle**		**ICC(3,1)**	**%95 CI**	
			**Lower**	**Upper**
**Transversus Abdominis**	Right	0.866	0.551	0.965
	Left	0.914	0.694	0.978
**L2–3 Multifidus**	Right	0.899	0.648	0.974
	Left	0.927	0.735	0.981
**L3–4 Multifidus**	Right	0.923	0.723	0.980
	Left	0.909	0.679	0.977
**L4–5 Multifidus**	Right	0.893	0.629	0.972
	Left	0.901	0.654	0.974

ICC: Intraclass Correlation Coefficient; CI: Confidence Interval; L2–3, L3–4, L4–5: Lumbar vertebral levels.
